# Impact of macronutrients and salinity stress on biomass and biochemical constituents in *Monoraphidium braunii* to enhance biodiesel production

**DOI:** 10.1038/s41598-024-53216-8

**Published:** 2024-02-01

**Authors:** Mostafa M. El-Sheekh, Hamdy R. Galal, Amal SH. H. Mousa, Abla A. M. Farghl

**Affiliations:** 1 Botany Department, Faculty of Science, Tanta University, Tanta, 31527 Egypt; 2 Botany and Microbiology Department, Faculty of Science, South Valley University, Qena, 83523 Egypt

**Keywords:** Energy, Industrial microbiology

## Abstract

Microalgal lipids are precursors to the production of biodiesel, as well as a source of valuable dietary components in the biotechnological industries. So, this study aimed to assess the effects of nutritional (nitrogen, and phosphorus) starvations and salinity stress (NaCl) on the biomass, lipid content, fatty acids profile, and predicted biodiesel properties of green microalga *Monoraphidium braunii*. The results showed that biomass, biomass productivity, and photosynthetic pigment contents (Chl. a, b, and carotenoids) of *M. braunii* were markedly decreased by nitrogen and phosphorus depletion and recorded the maximum values in cultures treated with full of N and P concentrations (control, 100%). These parameters were considerably increased at the low salinity level (up to 150 mM NaCl), while an increasing salinity level (up to 250 mM NaCl) reduces the biomass, its productivity, and pigment contents. Nutritional limitations and salt stress (NaCl) resulted in significantly enhanced accumulation of lipid and productivity of *M. braunii*, which represented more than twofold of the control. Furthermore, these conditions have enhanced the profile of fatty acid and biodiesel quality-related parameters. The current study exposed strategies to improve *M. braunii* lipid productivity for biodiesel production on a small scale in vitro in terms of fuel quality under low nutrients and salinity stress.

The increased demand for energy around the world resulted in higher oil prices and declining fossil fuel reserves, as well as problems with the environment and people’s health brought on by toxic pollutants. These problems have stimulated researchers to hunt for alternative, sustainable, eco-friendly, and renewable energy sources^1,2^. Bioenergy is a critical component for reducing emissions of greenhouse gas and replacing fossil fuels^3^. The primary fossil-derived energy sources up until now were natural gas and crude oil. The most well-established energy sources are hydro, nuclear, solar, wind sea waves, and fossil fuels (crude oil, natural gas, and coal) ^4^. The pollution caused by the use of petroleum diesel is the main disadvantage of using petroleum-based fuels. The burning of petroleum diesel contributes significantly to greenhouse gas emissions (GHG). Petroleum diesel is the main source of NOx, SOx, CO, particulate material, and volatile organic compounds as a source of air contaminants, in addition to these emissions^5^. Biodiesel has a lot of attention as a green fuel which environmentally friendly. Currently, agricultural plants are used as feedstock for biofuel puts them in direct competition with food production for freshwater and land, raising serious sustainability issues^6^.

Microalgae used as a third-generation biofuel has several benefits compared to first and second-generation biofuel, which are made from food crops and non-food wastes, respectively. For instance, microalgae can grow quickly (up to 20–30 times faster than oil crops), fix CO_2_ to increase biomass and O_2_ production, have a high lipid content, can grow on non-agricultural soils utilizing sewage or seawater, and can be collected all year^7^. Because of their lipid accumulation capacity and their strong photosynthetic productivity, algae produce up to 31 times more oil annually than palm oil^1^. Microalgae-based biodiesel is a promising sustainable energy source that can replace fossil fuels while preserving the supply of food resources for human consumption^2^. Microalgae lipids are a safe alternative to petroleum diesel. Because of their environmental friendliness, biodegradability, toxicity freeness, and low greenhouse gas (GHG) emissions. In addition to having a high lipid content, typical microalgae for biodiesel production also need to have a reasonable fatty acid composition. Microalgae produce saturated and monounsaturated fatty acids, which are perfect for producing biodiesel because they balance out cetane number and cold flow characteristics^8^. Many microalgae change their fat biosynthetic pathway in response to adverse environmental or stress conditions, resulting in the formation and accumulation of neutral lipids, primarily Triacylglycerides (TAGs), which are the greatest appropriate candidates for biodiesel synthesis^9^. *Monoraphadium* alga has been found through extensive screening of microalgae strains to be promising in terms of producing high biomass and increasing lipid yields when grown under stressful conditions. It was determined that the green algae *Monoraphidium* would make an excellent feedstock for biodiesel^10^. Stress conditions such as nutrient limitations, salt stress, light intensity, pH, and temperature showed a significant effect on microalgal growth and metabolism, including accumulation of lipids in microalgae^11^. TAG yield has been shown to increase in several microalgae when nutrients are limited or depleted, particularly nitrogen and phosphorus. Nutrient deficiency has a significant impact on lipid accumulation in algal cells^11^. Nitrogen is a component of peptides, energy transfer molecules, genetic materials, chlorophylls, and enzymes found in algal cells, in addition to structural and functional proteins^12^. Nitrogen deficiency in algal taxa can in a variety of responses, including protein reduction, variations in the photosynthetic pigment content, and an increase in lipid productivity^13^.

Phosphorus is a crucial abiotic macronutrient for algal growth. Its presence is essential for the synthesis of proteins, nucleic acids, and cellular membranes. Phosphorus deficiency can cause fluctuations in chlorophyll and protein content^14^. According to Almutairi^15^, lowering phosphorus concentrations by 50% increased the lipid content of *Dunaliella salina* (16.45%) by about threefold, whereas lowering the phosphorus concentration to zero (0.0P) increased the content of lipid by more than fourfold, to 24.86% when compared to the control culture (5.88%). Furthermore, while the restriction of the nitrogen had a greater impact on the accumulation of the lipids than phosphate restriction, limiting both nutrients concurrently increased lipid accumulation synergistically^16^.

Salinity has an effect on lipid accumulation in the microalgal cell as well. The ability of microalgae to adapt to salt stress varies according to their tolerance level. NaCl concentrations were increased from 13 to 40 g L^-1^ increased the total fatty acids (TFA) content of *Nannochloropsis* sp. At the lowest salinity level, eicosapentaenoic acid (EPA) production was slightly higher^17^.

According to Guschina and Harwood^18^, not all of the oils that algae produce can be used to make biodiesel. For example, biodiesel with a high content of saturated fatty acids (SFAs) has a higher thermal efficiency than biodiesel with a high content of unsaturated fatty acids (USFA), which releases more nitrogenous oxides and has a lower thermal efficiency^19^. Furthermore, it was discovered that the fuel properties of biodiesel were influenced by the fatty acids and alcohols present in fatty acids methyl esters (FAMEs)^20^. Additionally, some microalgal species have better fatty acid profiles, and the produced biodiesel has higher oxidation stability (OS) due to the unsaponified fraction and satisfies biodiesel standards^21^. Biodiesel created by pyrolysis has analogous fuel properties like high cetane number, low viscosity, acceptable corrosion rate for copper and sulfur concentration, and water and sediment content is within limits. However, the carbon residues, ash content, and pour points of the product are undesirable^22^.

In order to increase the synthesis of lipids for biodiesel production, this study aimed to evaluate how the green microalga *M. braunii* responded to nutrient limitation and salinity stress. In addition, the fatty acids profiles were explored to assess the qualities of biodiesel.

## Results and discussion

### Microalgal growth under nutrient deficiency and salt stress

**Figure 1:**
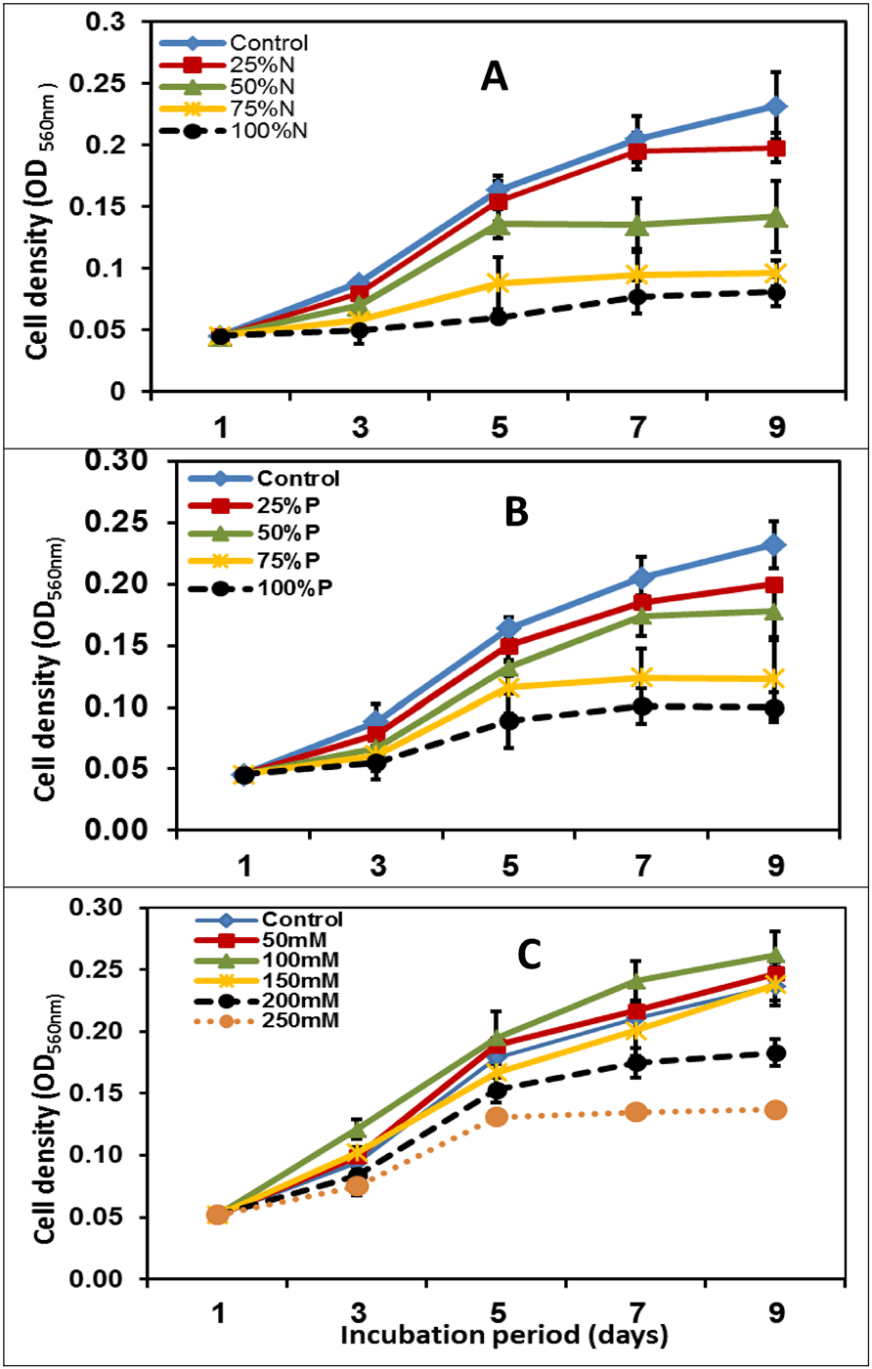
The growth curve of green microalga *M. braunii* grown in nitrogen (**A**) and phosphorus (**B**) deficiency and salt stress (**C**). Data are means of replicates with standard deviation (SD).

The growth rate of green alga *Monoraphidium braunii* grown in media containing various nitrogen and phosphorus concentrations is shown in Fig. 1A,B.

The growth rate was estimated using optical density (OD_560_) every two days, the highest growth of *M. braunii* was detected in cultures accompanied full of nitrogen and phosphorus concentrations (control, 100%), as a result, OD_560_ value was (0.232) at the 9th day of cultivation. On the other hand, the depletion of nitrogen and phosphorus concentrations resulted in a significant decrease in the growth rate of *Monoraphidium braunii*. The highest reduction in the growth rate of *M. braunii* cultured in medium containing (0.0 N) was 65.09%, whereas 56.90% in medium containing (0.0 P) as compared to the control at the 9th day of cultivation.

Almutairi^15^ revealed similar results using *Dunaliella salina* under nitrogen and phosphorus limitations*,* reporting that nutrient depletion induces growth failure. Similarly, reduced growth of *Chlamydomonas reinhardtii* has been found under N deficiency, even after the required phosphate addition^23^.

Regarding salinity stress, the growth rate (OD_560_) of green microalga *M. braunii* was improved by increasing the salt concentrations up to 150 mM NaCl, where it increased by 10% over the control (0 NaCl) at 100 mM NaCl, after that, significantly decreased was shown, it decreased by 42.19% at 250 mM NaCl compared to the control at 9th day of cultivation (Fig. 1C). Our results agreed with Pandit et al.^24^ revealed that growth rate of *Chlorella vulgaris* and *Acutodesmus obliquus* increased by increasing the concentration of NaCl from 0 to 0.1 M and decreased by increasing the dosage from 0.3 to 0.4 M in addition El-Sheekh et al.^25^ demonstrated that the addition of NaCl enhanced optical density of *S. obliquus* when compared to the control. From that perspective, salt stress has been shown to significantly affect growth while interfering with the physiological activities of algal cells. The salt tolerance level and species of microalgae determine their adaptability to saline conditions^26^. On the contrary, Alsull and Omar^27^ recorded that lower salinity conditions significantly reduced the optical density and dry weight of *Tetraselmis* sp. and *Nannochloropsis* sp.

### Biomass yield and biomass productivity under nutrient deficiency and salt stress

**Figure 2:**
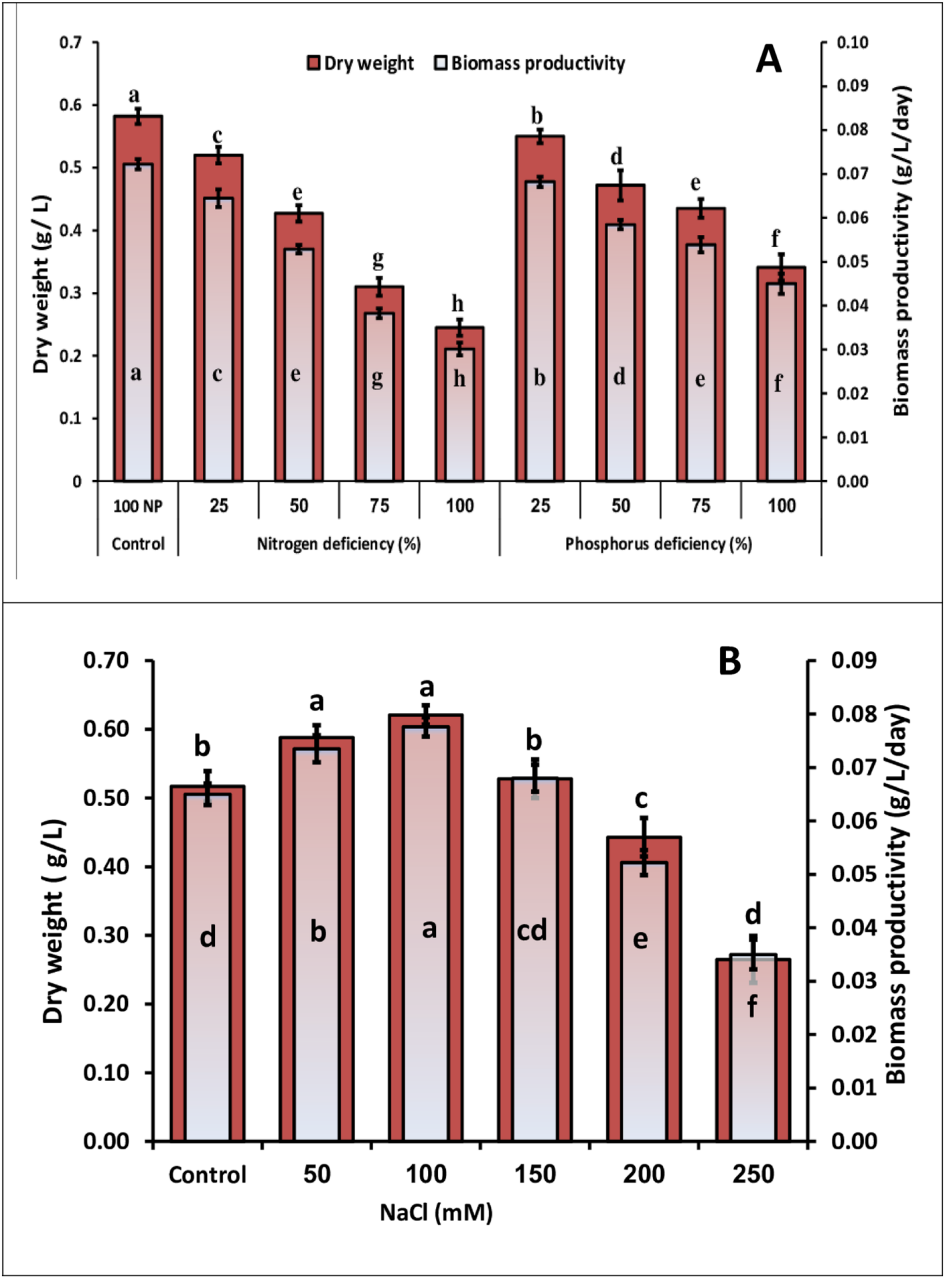
Biomass and biomass productivity of green microalga *M. braunii* grown in nutrients deficiency (**A**) and salt stress (**B**). Data are means of replicates with standard deviation. Means followed by different letters are significantly different according to Duncan’s multiple range comparison (DMRTs), and the same letters are not significantly different.

The cellular dry weight (CDW) and biomass productivity were measured on the last day of culture (9th day). Because they indicate how quickly different microalgal strains grow, biomass yield and productivity are essential factors in the production of biofuel^28^. The most important species for significant biomass production are those with faster growth rates and higher biomass productivity^29^. Biomass yield and the productivity of *M. braunii* significantly reduced (at p ≤ 0.05) as the nitrogen concentration was decreased (Fig. 2A). The highest decrease in biomass yield and its productivity of 0.245 g/L and 0.030 g/L/d, respectively was observed in 100% N deficiency culture as compared to the control (0.582 g/L and 0.072 g/L/d, respectively), with a reduction of 42% and 41%, respectively lower than control. In line with these results, the nitrogen deficiency in the synthetic media caused considerable reductions in dry weight and biomass productivity^30^. Nitrogen plays an important role in photosynthesis and contributes to the creation of numerous structural and functional molecules, including proteins, enzymes, and nucleic acids, which can be attributed to the high biomass yield^31^.

Dry weight and biomass productivity of *Monoraphidium braunii* in the late exponential phase markedly decreased under phosphorus depletion, especially in 100% of phosphorus deficiency culture, with a reduction of 58% and 62% compared to the control (100% P), respectively (Fig. 2A). In this respect, Xin et al.^32^ showed that phosphorus starvation reduced the total biomass of *Scenedesmus* sp. These findings revealed that nitrogen depletion had a greater effect on *M. braunii* growth than phosphorus depletion. On the other hand, the deficiency of nitrogen and phosphorus caused the fixing of carbon to lipids or carbohydrates synthesis. As a result, N and P stress conditions are regarded as the most critical for lipid metabolism^33^.

Concerning salt stress, dry weight and biomass productivity of *M. braunii* were considerably increased up to 150 mM NaCl. In culture treated with 100 mM NaCl, the highest dry weight and biomass productivity were observed (0.62 g L^-1^ and 0.078 g L^-1^ d^-1^, respectively ), with an increase of 20% and 19%, respectively when compared to the control followed by 50 mM NaCl (0.588 g L^-1^ and 0.074 g L^-1^d^-1^, respectively) corresponding (0.517 and 0.065 g L^-1^d^-1^, respectively) in the control. While increasing salinity levels reduces the biomass and its productivity, the highest percentage reduction in biomass and biomass productivity was 48.74 and 46.15%, respectively less than the corresponding control after 9 days of incubation at 250 mM NaCl (Fig. 2B). Low concentrations of NaCl are beneficial for some metabolic processes and promote microalgae growth. On the other hand, high levels of NaCl concentrations may cause inhibition of growth and death of cells^34^, these findings were consistent with our research. In contrast, El-Sheekh et al.^2^ reported that 300mM NaCl exhibited the greatest growth of *Microchloropsis salina* as compared to other salinity concentrations. Normal salt stress affects phytoplankton through (1) mechanisms of ion homeostasis (2) alterations in cellular ionic ratios caused by selective ion permeability in the membrane, (3) Osmolytes are classified into types: osmolytes and osmoprotectants^35^. Microalgae have unique mechanisms for adjust with salinity stress. Mechanisms may involve accumulating osmo-protective solutes, producing antioxidant enzymes, regulating ion exchange processes, and shifting from active cell division to energy storage through lipids. As a result, microalgae can tolerant salinity stress and grow more efficiently^36^.

### Photosynthetic pigments under nutrient deficiency and salt stress

**Figure 3:**
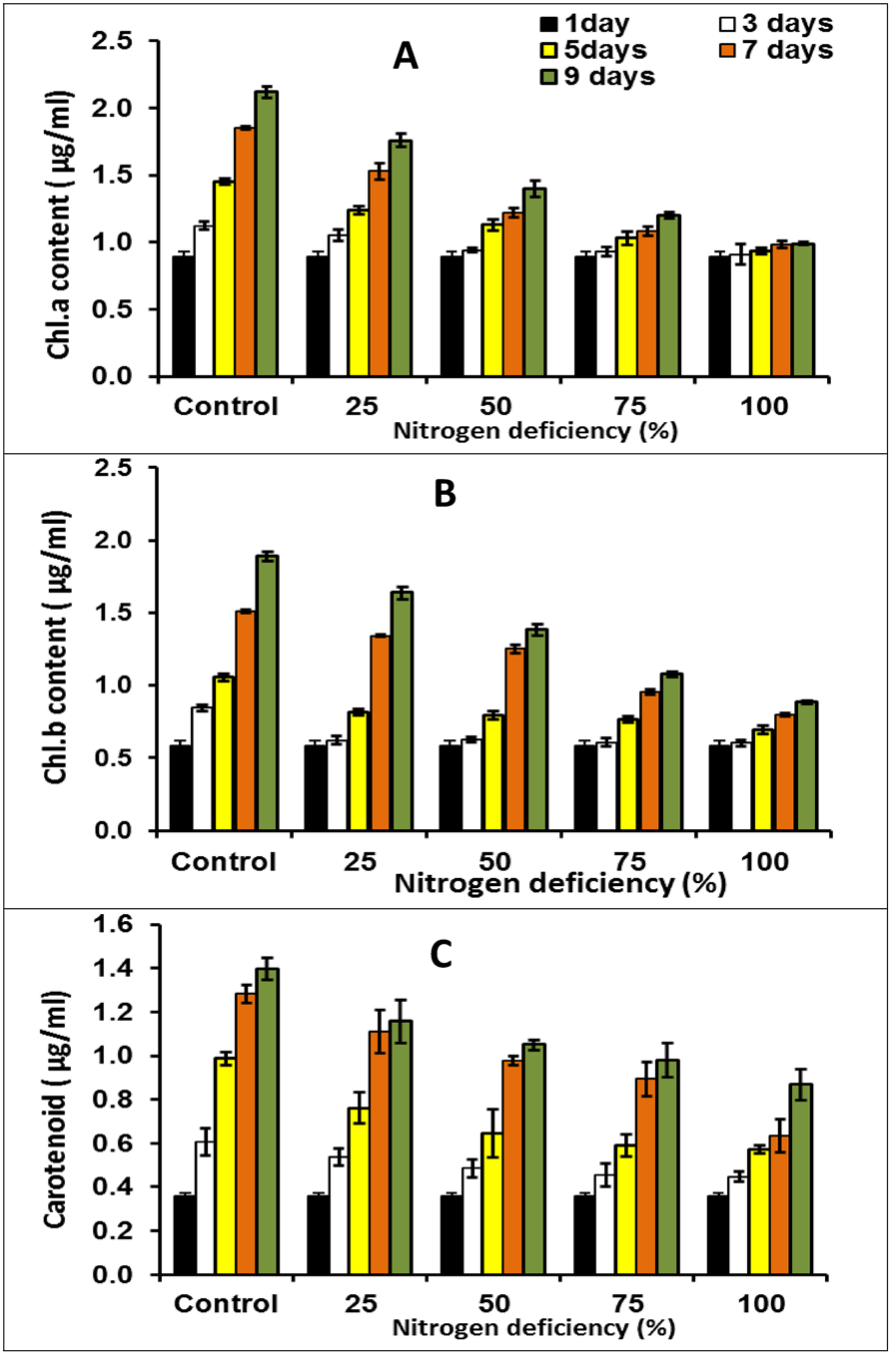
Chl.a, (**A**) Chl.b (**B**) and carotenoid (**C**) contents of green microalga *Monoraphidium* *braunii* grown under nitrogen deficiency. Data are means of replicates with standard deviation.

During this study, the pigments, chlorophyll (Chl.) a, b, and carotenoids were assessed every two days which could be used as a biomass criterion and an indicator for the growth of microalgae. The results in Fig. 3 revealed that exposure of *M. braunii* cultures to the reduction in phosphorus concentration was accompanied by a significant decrease in photosynthetic pigment contents (Chl. a, b, and carotenoids).

**Figure 4:**
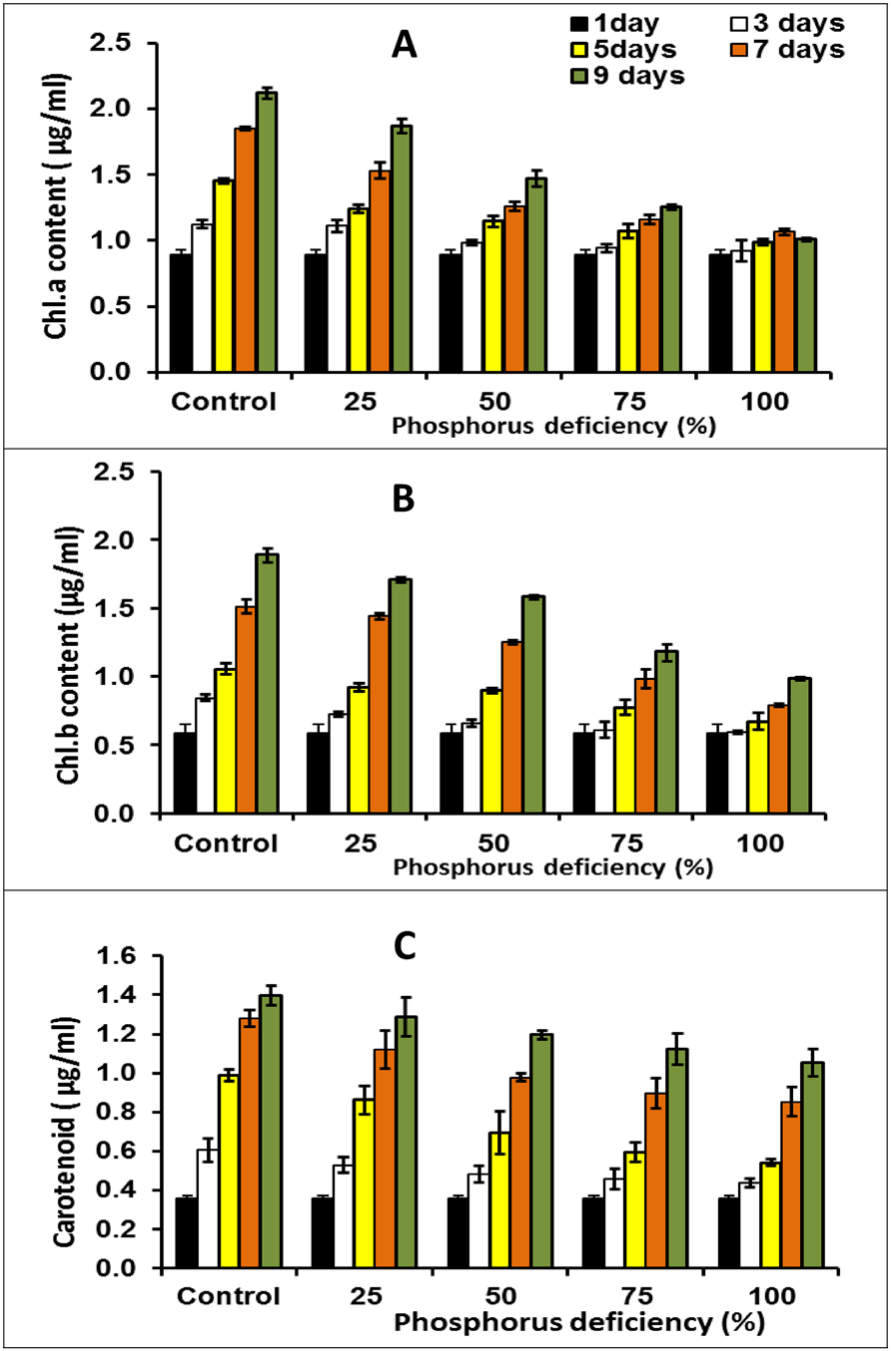
Chl.a, (**A**) Chl.b (**B**) and carotenoid (**C**) contents of green microalga *Monoraphidium* *braunii* grown under phosphorus deficiency. Data are means of replicates with standard deviation.

100% nitrogen deficiency caused Chl. a, b, and carotenoids decrease by 46.74, 47.04, and 62.35%, respectively contrasted with control (100% P). A similar trend was observed in the results of phosphorus reduction cultures (Fig. 4), where the Chl. a, b, and carotenoid contents decreased by 47.64, 52.27, 75.44%, respectively, after 9 days of cultivation. Phosphorus is a vital abiotic macronutrient for the development of cellular structure, nucleic acid synthesis, algal growth, and protein synthesis. Low protein content and chlorophyll destruction are both consequences of phosphorus deficiency^37^. According to Breuer et al^38^, nitrogen deficiency causes a reduction in protein, which results in a decrease in chlorophyll content in microalgae cells. This apparent reduction in chlorophyll content could be explained by nitrogen restriction on the photosynthetic system within algal cells^30^. In this regard, Xin et al.^32^ stated that phosphorus starvation reduced the chlorophyll content of *Scenedesmus* sp.

**Figure 5:**
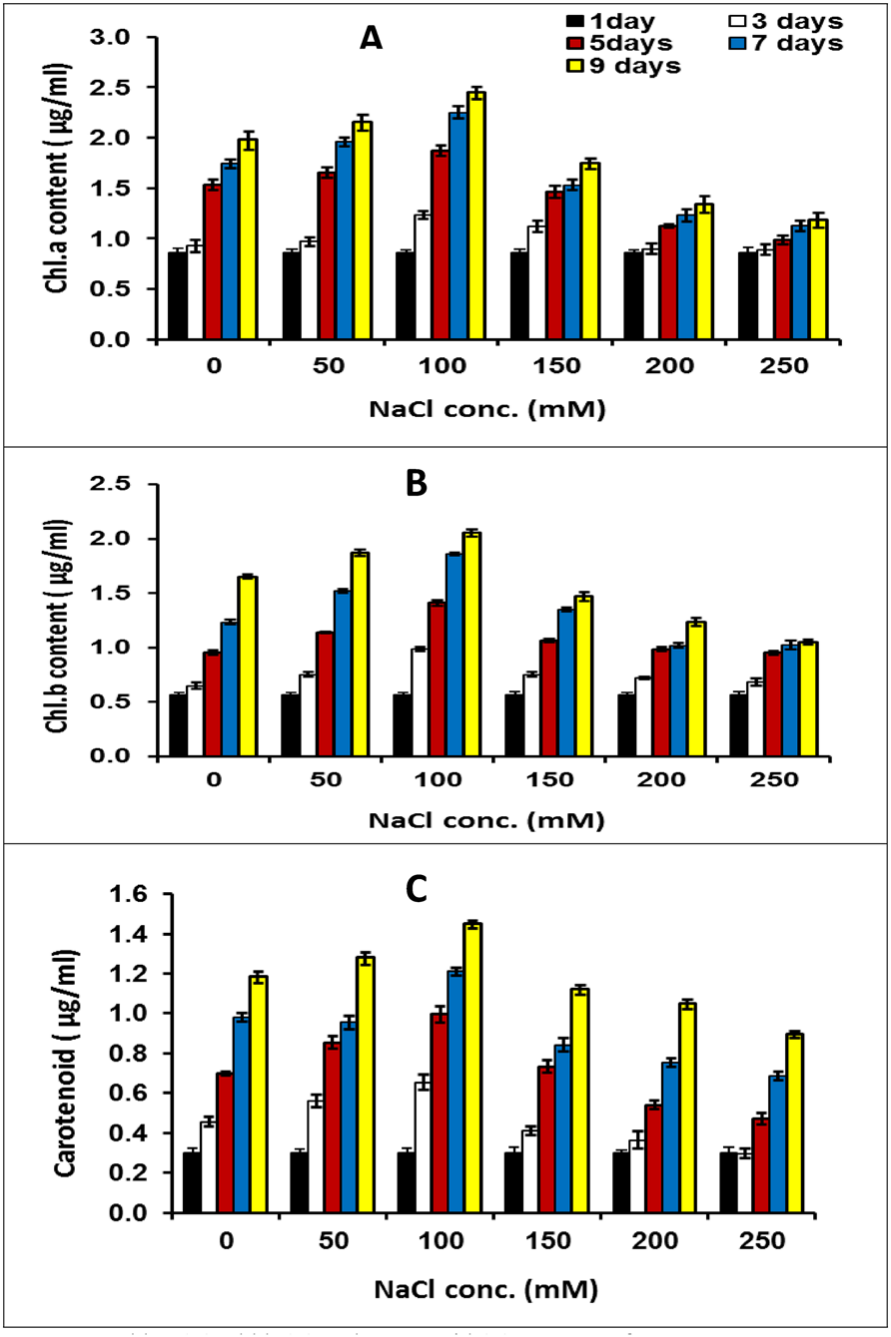
Chl.a, (**A**) Chl.b (**B**) and carotenoid (**C**) contents of green microalga *Monoraphidium* *braunii* grown under different concentrations of salt stress (NaCl). Data are means of replicates with standard deviation.

Salt toxicity limits the rate net of CO_2_ assimilation, which lowers the chlorophyll (Chl) and growth^39^. The content of photosynthetic pigments, Chl a, Chl b and carotenoids of *M. braunii* were measured every two days are revealed here (Fig. 5). Chl. a, Chl. b, and carotenoids of *M. braunii* were considerably increased with increasing salinity up to 100 mM NaCl (23, 24 and 22% over the control, respectively). The maximum percentage reduction in Chl. a, Chl. b, and carotenoids was recorded 40%, 36% and 24%, respectively with 250 mM NaCl compared to the control on the 9th day of cultivation (Fig. 5). These results were consistent with those of Fal et al.^40^, who found that at 0 and 200 mM NaCl, Chl a, and Chl b levels of *C. reinhardtii* were significantly decreased from 7.15 mg L^-1^ and 1.67 mg L^-1^ to 5.28 mg L^-1^ and 1.24 mg L^-1^, respectively. Pandit et al.^24^ discovered similar results using *Chlorella vulgaris* under salinity stress, reporting that low salinity dose increased photosynthetic pigments and decreased at higher concentrations. On the contrary, Pandit et al.^24^, using *Acutodesmus obliquus* under salinity stress, found that low salinity dose reduced photosynthetic pigments. Higher salinities cause lower chlorophyll contents due to osmotic and toxic ionic stress, which reduces the photosynthesis rate and thus lowers chlorophyll and protein content^29^. Chl a deficiency may be a sign of oxidative stress brought on by increased chlorophyllase activity, which promotes the degradation of Chl^41^. Also, it could be related to a decrease in Rubisco activity as a result of the low CO_2_ uptake^39^.

### Lipid content and lipid productivity of *M*. *braunii* under nutrient deficiency and salt stress

**Figure 6:**
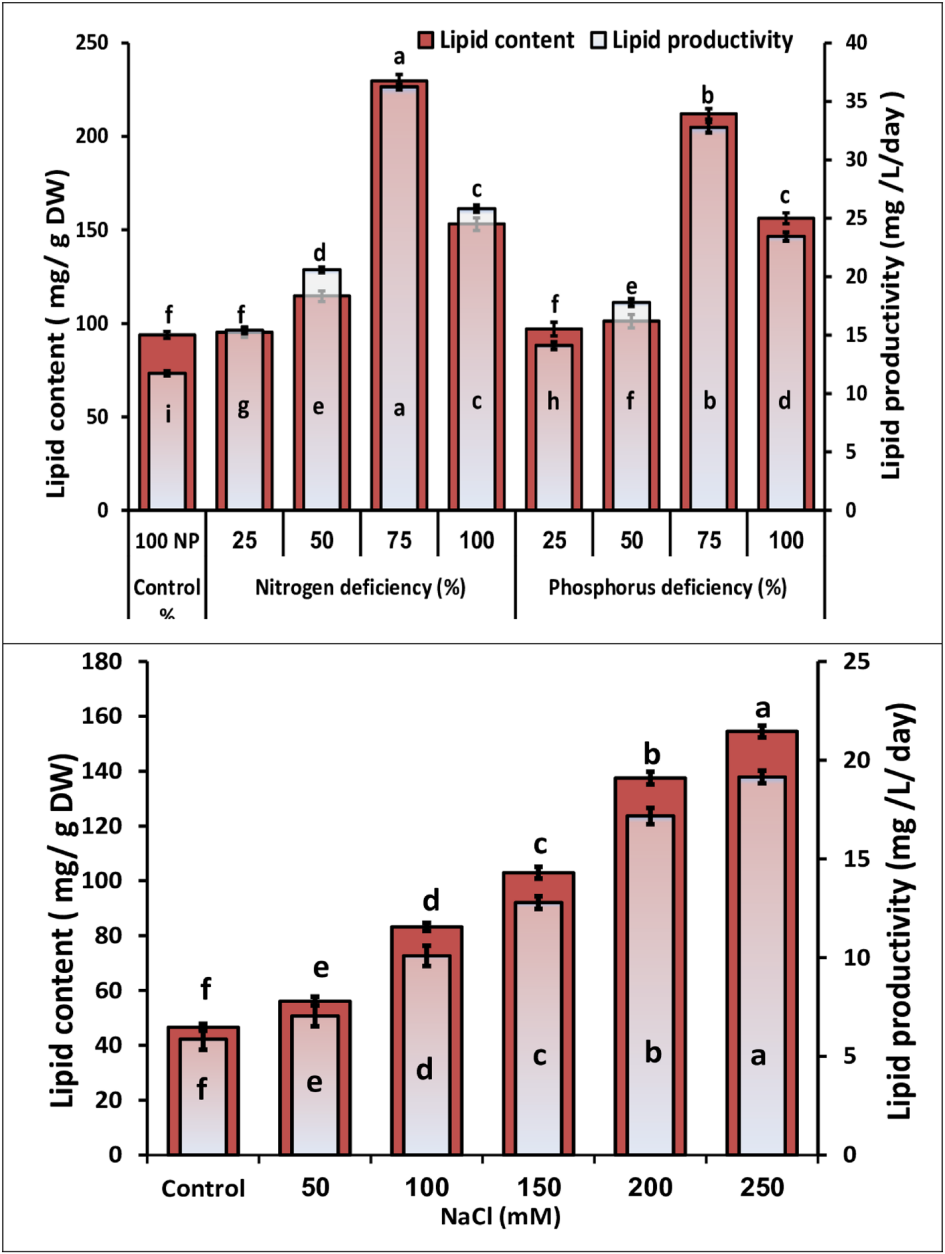
Lipid content and lipid productivity of green microalga *M. braunii* grown in nutrients deficiency (**A**) and salinity stress (**B**). Data are means of replicates with standard deviation. Means followed by different letters are significantly different according to Duncan’s multiple range comparison (DMRTs), and the same letters are not significantly different.

Lipid content of *M*. *braunii* increased significantly during cultivation under nitrogen and phosphorus depletion, reaching a maximum of 229.63 and 212.09 mg g^-1^ at 75% nitrogen and phosphorus deficiency, respectively, which represented more than twofold of the control (Fig. 6). The increase in lipid content in algal cells in response to nitrogen deficiency is thought to be a defense mechanism to prevent protein breakdown and a decline in growth dry weight^42^. The reduction of N and P was effective for the lipid productivity of *M*. *braunii* as shown in Fig. 6. A 75% reduction in nitrogen and phosphorus was the most effective condition, where lipid productivity increased by more than threefold and twofold (up to 209 and179%, respectively) of the control grown under 100% nitrogen (N–NO_3_^-^) and phosphorus (P-PO_4_^3-^), followed by sever nitrogen (100% deficiency) induced lipid productivity up to 2.2 fold of the control for *M*. *braunii*. These findings revealed that decreasing nitrogen concentration had a greater effect on *M*. *braunii* lipid accumulation and productivity than phosphorus concentration reduction. These results are in agreement with other studies that investigated the effects of the reduction in nitrogen and phosphorus concentrations on lipid production^15,43^. In general, nitrogen concentration has a significant effect on the biochemical compositions and growth of microalgae; conversely, nitrogen depletion in culture medium results in a reduction in growth with concurrent enhanced lipid productivity^44^. Nitrogen starvation is an effective method for increasing lipid production in microalgal cells. Lipids produced in the case of nitrogen deficiency are two-fold those produced in the case of nitrogen-sufficient medium ^45^. Phosphorus plays a critical role in the transfer of energy. In the deficiency, it increases the lipid accumulation of algae. Nutrient deficiency (i.e., nitrogen and phosphorus limited) for algal cells is not unusual in the natural environment, where nutrient limitation shifts the organism’s metabolic pathway. For instance, the metabolism of lipids changes from neutral lipid storage to membrane lipid synthesis when nitrogen and phosphorus are deficient, as a result, the total lipid content of green algae increase^46^.

Concerning saline stress, the lipid content and lipid productivity of *M. braunii* increased significantly as the NaCl concentration increased from 50 to 250 mM, increasing more than threefold of the control at 250 mM NaCl (Fig. 6).

Numerous studies have found that unfavorable conditions, such as nutrient deficiency or high salinity, promote higher lipid levels^40,47^. These results are in agreement with another study in which the lipid content in *Botryococcus braunii* grown in 0.50 M NaCl was higher than those without NaCl^48^. Hang et al.^49^ found that a high NaCl concentration (200 mM) significantly increased lipid content (41.1%) in *C. reinhardtii* compared to the control (20.8%) after 3 days of application. To increase the production of lipids and value-added products, microalgae's stress tolerance and environmental stress tolerance are frequently altered^50^.

According to Fal et al.^40^, the content of lipids also increased significantly, rising from 9.35% and 27.18% under 0 and 200 mM NaCl conditions, respectively. This lipid accumulation, specifically neutral lipids, reduces the osmotic pressure and fluidity of cell membranes, which helps to maintain the membrane integrity in response to salt stress^41^. According to Dahmen et al.^51^, a high salinity growth medium increased microalga cellular content by enhancing TAG formation. The most effective microalgae for producing biodiesel are essentially determined by their lipid accumulation and lipid productivity both of which are influenced by biomass production^31^^.^

### Gas chromatography (GC) analysis of fatty acids

**Figure 7:**
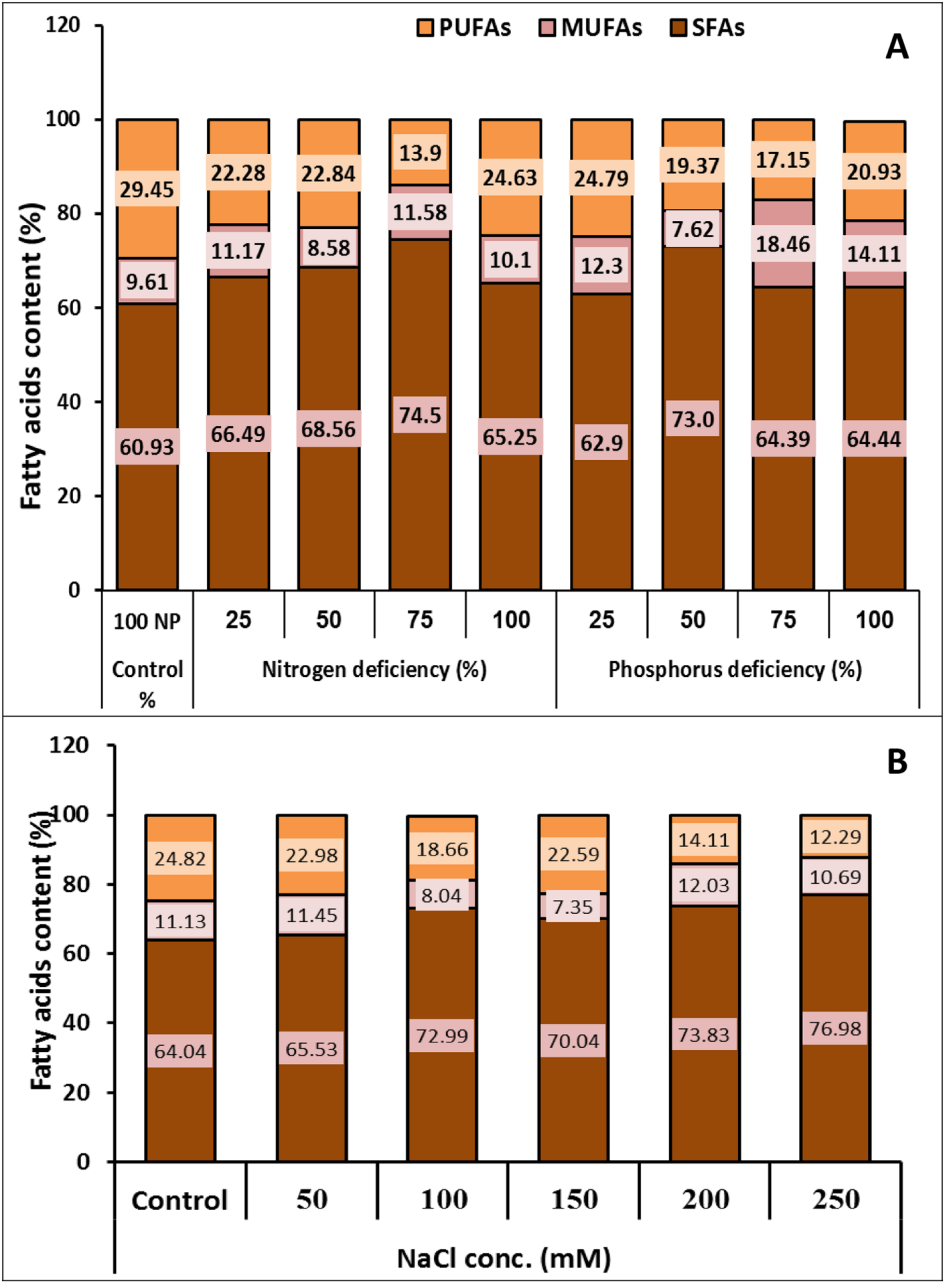
Fatty acids composition (%) of green microalga *M. braunii* grown in nutrients deficiency (**A**) and salt stress (**B**).

Characteristics of fatty acid profiles determine whether a lipid is suitable for biodiesel production even if lipid content and productivity are important factors in determining the best conditions for biofuel production^52^. As a result, the current study included a complete analysis of fatty acid composition. The fatty acid profile of *Monoraphidium braunii* showed an increase in saturated fatty acids (SFAs) C14:0, C16:0, and C18:0 and monounsaturated fatty acids (MUFAs) C16:1 and C18:1 while exhibiting a decrease in in in polyunsaturated fatty acids (PUFAs) C18:2 and C18:3 under reduced nitrogen and phosphorus concentrations, as well as salinity stress when compared to the control conditions (Table 1 and Fig. 7). Saturated fatty acids (SFAs) exhibited the maximum proportion, which varied from 60.93 to 76.98% of total fatty acids (TFAs), followed by polyunsaturated fatty acids (PUFAs) (12.29 to 29.45%) and monounsaturated fatty acids (MUFAs) (7.62% to 18.46%) in *Monoraphidium braunii*. The biomass produced the highest percentage of SFAs (76.98%) under 250 mM NaCl followed by 75% of nitrogen deficiency (74.5%), in excess of 20% of biomass produced in the control. The saturated fatty acids, palmitic acid (C16:0) and stearic acid (C18:0) were the most abundant fatty acids in the *M. braunii* oil biodiesel under different conditions. Monounsaturated fatty acids (MUFAs) in the *M. braunii* were represented by oleic acid (C18:1) and palmitoleic acid (C16:1), oleic acid was the major monounsaturated fatty acid that was found in *M*. *braunii* oil biodiesel where the highest value 15.85% of total fatty acids at 75% of phosphorus deficiency. Polyunsaturated fatty acids in the *M. braunii* showed the highest value (29.45%) at the control condition (100% N and P) and the highest decrease by 41% of the control at 250 mM NaCl. These data agree with Sukkrom^53^ reported that the C_16_ and C_18_ fatty acid groups accounted for more than 80% of the total fatty acids in microalgae cultured in all media. Also, Widianingsih et al.^54^ stated that the most common fatty acids found in biodiesel were palmitic, stearic, oleic, and linolenic acids. These results were also consistent with Dahmen et al.^51^ who observed that salinity stress increased total SFAs and MUFAs while decreasing PUFAs in microgreen algae. Variations in the fatty acid profile in the medium as salinity levels rise are required to keep the membrane fluid intact and prevent its destruction^35^.

**Table 1:** Fatty acid profile of *M*. *braunii* cultivated in nutrient deficiency and salt stress.

Fatty acid methyl esters	Control	Nitrogen deficiency (%)				Phosphorous deficiency (%)				NaCl Conc.(mM)					
SFA		25	50	75	100	25	50	75	100	0	50	100	150	200	250
Myristic acid C14:0	1.3	1.5	1.65	1.18	1.49	0.99	1.04	0.97	1.31	1.31	1.38	1.19	1.08	1.58	1.4
Palmitic acid C16:0	43.12	45.21	45.61	46.4	42.74	43.79	45.21	45.36	44	45.24	47.39	46.4	46.16	46.36	49.41
Stearic acid C18:0	16.51	19.78	21.3	26.92	21.02	18.12	26.75	18.06	19.13	17.49	16.76	25.4	22.8	25.89	26.17
Ʃ SFA	60.93	66.49	68.56	74.5	65.25	62.9	73	64.39	64.44	64.04	65.53	72.99	70.04	73.83	76.98
MUFA															
Palmitoleic acid C16:1	1.97	2.41	2.15	2.7	2.03	2.33	1.82	2.61	2.75	2.73	1.52	1.31	1.64	1.72	2.34
Oleic acid C18:1	7.64	8.76	6.43	8.88	8.07	9.97	5.8	15.85	11.36	8.4	9.93	6.73	5.71	10.31	8.35
Ʃ MUFA	9.61	11.17	8.58	11.58	10.1	12.3	7.62	18.46	14.11	11.13	11.45	8.04	7.35	12.03	10.69
PUFAs															
Benzene propanoic acid C17:3	6.81	8.1	7.21	6.36	8.25	7.25	8.63	7.73	7.3	8.34	7.45	9.75	9.46	6.33	6.66
Linoleic acid C18:2	16.45	11.1	11.8	5.88	11.8	10.22	7.5	6.05	7.2	10.23	10.63	6.17	8.89	4.94	4.12
Linolenic acid C18:3	6.19	3.08	3.83	1.66	4.58	7.32	3.24	3.37	6.43	6.25	4.9	2.74	4.24	2.84	1.51
Ʃ PUFAs	29.45	22.28	22.84	13.9	24.63	24.79	19.37	17.15	20.93	24.82	22.98	18.66	22.59	14.11	12.29
Ʃ total FAs	99.99	99.94	99.98	99.98	99.98	99.99	99.99	99.97	99.48	99.99	99.96	99.69	99.98	99.97	99.96

SFA: Saturated fatty acids, MUFA: Monounsaturated fatty acids, PUFAs: polyunsaturated fatty acids.

The current study discovered that the FA profile changed with different conditions, resulting in fatty acid content variation. These findings were in line with those of Srinuanpan et al.^55^, which exposed that variation in the fatty acid profile caused by nutrient manipulation that was considered favorable for biodiesel quality.

The proportions of SFA, MUFA, and PUFA in the FAs compositions varied significantly under various circumstances (p ≤ 0.05) (Fig. 7). This might be a result of variation in lipid production and composition of fatty acid in response to various stressors^56^. Algal lipids had higher SFA levels than biodiesel's traditional soybean and canola oil feedstock^57^. Furthermore, microalgae with low growth rates in nitrogen and phosphorus-deficient conditions accumulate more saturated fatty acids (SFA) and monounsaturated fatty acids (MUFA) into neutral lipids. Contrarily, higher growth causes more accumulation of polyunsaturated fatty acids (PUFA)^58^. As a result, we can conclude that *Monoraphidium braunii* is a suitable producer of saturated fatty acids that are easily convertible to biodiesel under nitrogen and phosphorus deficiency as well as salinity stress.

### Biodiesel properties

Biodiesel properties are primarily determined by the fatty acids profile of the feedstock used. The most suitable fatty acid methyl esters (FAMEs) for high-quality biodiesel are those with longer chain lengths and lower levels of carbon chain unsaturation^59^. Biodiesel of *Monoraphidium braunii* which is cultivated in nitrogen and phosphorus medium starvation as well as salt stress had high saturated and low unsaturated fatty acid content, which is preferable for high-quality biodiesel because saturated fatty acids promote transesterification^60^. The values of unsaturation degree (Du), kinematic viscosity (KV), specific gravity (SG), cloud point (CP), cetane number (CN), iodine value (IV), saponification value (SV), higher heating value (HHV), Long-chain saturation factor (LCSF) and cold filter plugging point (CFPP) were estimated in present study (Table 2), were found to be in line with LCSF and CFPP in the international standards (ASTM D6751 and EN 14,214). The degree of unsaturation (DU) was calculated by multiplying double bonds C=C and the mass fraction of each individual fatty acid, and it mainly indicates the number of unsaturated FAs. The degree of unsaturation (DU) was decreased with nitrogen and phosphorus starvation as well as salt stress to reach to lowest value of 0.49% in the case of 200 mM NaCl. The high DU value of the biofuel indicated that it was more difficult to form deposits and had better lubricity^34^. The cetane number (CN) increases with increasing saturation level of fatty acid and represents the ignition quality of the biodiesel and it^61^. The results showed an increase in cetane number under different stresses where the highest value is 59.58 at 200 mM of NaCl compared to 58.98 at the control, followed by CN value 58.99 and 58.98 at 50% phosphorus and nitrogen deficiency, respectively compared to 57.44 in the control biodiesel which is due to the high content of palmitic acid (C16:0) and stearic acid (C18:0). These values confirm that the microalgae biodiesel has a high ignition quality and meets the lowest CN values prescribed by international EN-14214, ASTM D675140 biofuel standards, the Australian standard, and the Brazilian National Petroleum Agency. It was noted that the cetane number varies greatly depending on engine speed, the lower the engine speed, the lower the cetane number (as low as 20) of fuel it can use^62^. The high value of cetane number is often related to enhanced performance and cleaner fuel combustion. A high CN value is preferred for biodiesel production because it is proportional to oxidative stability and ignition quality and can be achieved at lower unsaturation levels^63^. There is a strong positive correlation between low NOx emissions and a high cetane number, which is a desirable characteristic in the production of clean fuel^64^. Considering that fossil diesel has a cetane number of 49.6^65^. Iodine value (IV) means the amount of iodine absorbed in 100 g of the fuel sample by double bonds of the FAME molecules. A higher iodine value indicates higher fats and oils, lower value of IV is favorable for biodiesel quality^17^, the results showed IV value was decreased with nitrogen and phosphorus starvation as well as NaCl to reach the lowest value of 49.51 (g I_2_.100 g^-1^ oil) in case of 200 mM of NaCl and IV found to be 56.02 and 56.14 (g I_2_.100 g^-1^ oil) at the depletion of phosphorus and nitrogen by 50%, respectively that proportionated to the USFAs^60^. The IV value of *M. braunii* with and without stress was lower than the maximum IV (120 g I2/100 g) of biodiesel prescribed by EN 14,214 standards. It should be noted that the biodiesel with the lowest content of unsaturated FAs has the lowest IV value. In previous studies, more or less similar lower values of IV were recorded for *C. vulgaris ranging* from 53.91 to 65.52 I_2_/100 g, and for *S. obliquus* ranging from 53.91 to 68.20 I_2_/100 g^55^.

**Table 2:** Biodiesel characteristics based on the fatty acids profile of* M*. *braunii* cultivated in nutrient deficiency and salt stress in comparison with the international standards.

Predicted biodiesl characteristics	Control	Nitrogen deficiency (%)				Phosphorus deficiency (%)				NaCl Conc.(mM)						International Standards of Biodiesl	
		25	50	75	100	25	50	75	100	Control	50	100	150	200	250	*ASTM D6751*#	EN 14,214*
DU	0.82	0.67	0.58	0.65	0.73	0.76	0.54	0.64	0.66	0.58	0.51	0.70	0.50	0.49	0.69	–	–
KV (mm^2^ s^−1^)	4.69	5.21	5.21	2.11	1.92	4.72	4.87	4.80	4.79	4.84	4.89	4.77	4.89	4.89	4.77	1.9–6.0	3.5–5.0
SG (kg^−1^)	0.88	0.88	0.88	0.88	0.88	0.88	0.88	0.88	0.88	0.88	0.88	0.88	0.88	0.88	0.88	0.85–0.9	-
CP (°C)	9.11	11.06	12.19	11.27	10.22	9.78	12.82	11.46	11.15	12.20	13.20	10.66	13.37	13.39	10.81	-	-
CN	57.44	58.41	58.98	58.52	58.00	57.78	58.99	58.62	58.46	58.98	59.48	58.22	59.57	59.58	58.29	≥ 47	≥ 51
IV (g I2.100 g^-1^ oil)	73.33	62.47	56.14	61.28	67.14	69.57	56.02	60.20	61.97	56.11	50.54	64.68	49.58	49.51	63.85	–	≤ 120
SV (mg KOH g^−1^)	208.97	209.81	209.83	209.79	209.29	209.24	209.37	209.53	209.79	210.59	209.94	209.98	210.34	209.46	209.63	–	–
HHV (Mj kg^−1^)	39.97	39.71	39.56	39.68	39.82	39.88	39.48	39.66	39.70	39.56	39.43	39.76	39.41	39.40	39.74	–	–
LCSF (wt %)	12.57	14.42	18.02	15.22	14.79	13.44	16.40	13.57	16.03	13.47	18.48	13.12	18.03	17.58	14.11	–	–
CFPP (°C)	23.00	28.82	40.12	31.33	29.98	25.74	35.03	26.14	33.87	25.84	41.59	24.74	40.16	38.76	27.85	− 13 to − 5	≤ 5/ ≤ − 20

DU: Degree of unsaturation, KV: kinematic viscosity, SG: Specific gravity, CP: Cloud point, CN: Cetane number, IV: Iodine value, SV: Saponification value, HHV: Higher heating value, LCSF: Long-chain saturation factor, CFPP: Cold filter plugging point.

ASTM International (formerly American Society for Testing and Materials)- EN 14,214, The European biodiesel specification.

The Saponification value (SV) is the amount of KOH (mg) required to saponify one gram of oil. The molecular weights of triglycerides are defined as SV in the oil. It is in reverse to the average molecular weight or the chain length of the fatty acids^66^. Thus, long-chain fatty acids are present in the lipids indicating a low SV. The limit of saponification value was not specified in biodiesel standards such as ASTM D6751, EN 14,214, and IS 15,607. The SV of *M. braunii* under nutrient deficiency and salt stress were 209.24–210.59 mg KOH/g oil, which is close to those of *M. reisseri* SIT04 (208.02–214.51 mg KOH/g oil) and *S. obliquus* SIT06 (210.95–216.73 mg KOH/g oil)^67^.

Low-temperature properties such as cold filter plugging point (CFPP) and cloud point (CP) of biodiesel were determined based on FA composition. Most of the treated cultures FAMEs showed an increase in CFPP and high values more than biodiesel standard EN 14,214 (− 13 to − 5 °C) and ASTM D6751(≤ 5/ ≤  − 20 °C) prescribed due to the decrease of polyunsaturated fatty acids (PUFAs) and high level of SFA, mainly the palmitic acid (C16:0) and stearic acid (C18:0), thus the higher proportion of these fatty acids will affect the low-temperature properties^68^. To confirm that a suitable fuel supply reaches injectors at various operating temperatures, biodiesel must have an appropriate kinematic viscosity (υ)^69^. Kinematic viscosity (υ), which has an inverse relationship with temperature, affects the engine's CFPP performance at low temperatures. According to ASTM6751-02 and en14214, the kinematic viscosity limits are 1.9–6.0 mm^2^ s^-1^ and 3.5–5.0 mm^2^ s^-1^, respectively. For *M. braunii* strains, the overall kinematic viscosity of the biodiesel fuel is within the range.

A specific cloud point (CP) value has not been specified by the biodiesel standards (ASTMD6751 and EN1424) because of the wide seasonal and temperature. In the present study, the results revealed that the determination cloud point for algal culture with different concentrations of nitrogen and phosphorus, as well as NaCl, increased under deficiency conditions where it ranged from 9.11 °C at the control to 12.82 °C at 50% P. This is in line with the previous studies that CP value of −2.47 to 9.71 °C^70^. A higher heating value (HHV) of biodiesel is an important property that indicates the amount of heat released by the fuel combustion of a unit quantity of fuel. The present results showed a lower HHV value under cultivation stress conditions except for 100 and 250 mM of NaCl exerts HHV more than control, where it ranged from 39.40 to 39.97 Mj kg^−1^. These values were acceptable according to the previous studies^56^, and they agree with earlier findings for *Anabaena variabilis* (39.87 Mj kg^−1^)^48^. Accordingly, its biodiesel properties produced by growing in the nitrogen and phosphorus starvation as well as salinity stress in this study can be applied to modern engines. In general, the results shown in Table 2 were maintained by the study, which asserted that most of the properties of biodiesel came from the species of microalga that were studied and met the limit values set by ASTM D6751 and EN 14,214:2012 + A2:2019 biodiesel standards^71^.

## Conclusion

It is worth noting that growing microalga *Monoraphidium braunii* in nutrient-restricted environments and saline stress has advantages for both the economy and the environment and is the recommended best approach for producing a lot of algal lipid content, productivity, and biodiesel. Furthermore, it was exposed that the properties of biodiesel of *Monoraphidium braunii* grown under these conditions are highly aligned with global biodiesel standards (ASTM D6751 and EN 14,214), assembly it is a strong contender for large-scale biodiesel production. According to the findings of this study, the native strain *M. braunii* could be considered a promising feedstock for sustainable energy production.

## Materials and methods

### Microalgae and growth conditions

The green alga *Monoraphidium braunii* was isolated for the first time from a water sample from South Valley University, south Egypt and grown in Erlenmeyer flasks of 500 ml in Beijerinck media^72^ under continuous illumination fluorescent light of 100 µmol m^−2^ s^−1^ at 25 °C and aerated with an air pump. The algal cultures were subjected to different deficiencies of nitrogen, and phosphorus (25%, 50%, 75%, and 100%, Table 3). Beijerinck media with 100% nitrogen and phosphorus concentrations were considered the standard treatment (control). Salinity stress was investigated using various concentrations of NaCl (50, 100, 150, 200, and 250 mM) added to the culture media, where the culture without NaCl was used as a control. Algal cells were added to the media to give a concentration of 5%. After 9 days of cultivation, the microalgal strains were harvested to determine and compare the biochemical analysis, fatty acids profile, and biodiesel properties.

**Table 3:** The concentrations of the different nutritional compositions of Beijerinck media (mg L^−1^).

Nutrients	Control (100%)	75%	50%	25%	0%
NH_4_NO_3_	1.5	1.125	0.75	0.375	0
K_2_HPO_4_	11.61	8.7075	5.805	2.9025	0
KH_2_PO_4_	9.07	6.8025	4.535	2.2675	0

### Determination of microalgal growth

#### Optical density

To estimate the growth of each algal culture, optical density at 560 nm (OD560) was determined using a spectrophotometer every two days. This wavelength was recommended for green algae by Wetherel^73^. Plot the growth curve of the respective algae cultures by using the absorbance obtained.

### Biomass yield and biomass productivity

Algal growth was measured also by determining the cellular dry weight (CDW) of algal species and biomass productivity. To measure the biomass productivity of *M. braunii*, the algal suspension (30 mL) was centrifuged at 4000 rpm on the last day of culture for 15 min, followed by several thorough washes with distilled water to remove trace of the growth medium, then dried overnight at 60 °C until constant weight. Biomass productivity was calculated using Eq. (1), as discussed by Abomohra et al.^74^.

1 $${\text{Biomass productivity BP }}\left( {{\text{g L}}^{{ - {1}}} {\text{day}}^{{ - {1}}} } \right) = \left( {{\text{CDW}}_{{\text{L}}} - {\text{CDW}}_{{\text{E}}} } \right)/\left( {{\text{t}}_{{\text{L}}} - {\text{t}}_{{\text{E}}} } \right)$$

where CDW_E_ and CDW_L_ represent the cellular dry weight (CDW) at the early exponential phase (t_E_) and at the time of the late exponential phase (t_L_) as g/L, respectively.

### Photosynthetic pigment

The pigment contents (Chl.a, Chl.b, and carotenoids) were assessed using a spectrophotometer every two days in agreement with the method of Metzner et al.^75^. Pigment fractions were calculated by µg/ ml algal suspension.

### Determination of total lipids and productivity

To extract total lipids from algae, El-Sheekh et al.^76^ modified protocol was used. Cells were homogenized in a 2:1 mixture of chloroform and methanol. After an orbital shaker the mixture was stirred at room temperature for 20 min. 0.2 ml of 0.9% sodium chloride solution was used to wash the solvent. The homogenate was separated into two phases using centrifugation. The lipid extracts were dried in 30 min at 80 °C, an argon stream, in pre-weighted glass vials, cooled in a desiccator, and then weighed. Andarad and Costa^77^ (Eq. 2) were used to determine lipid productivity, which was modified by Abomohra et al.^74^.

2 $${\text{Lipid productivity }}\left( {{\text{LP}},{\text{mg L}}^{{ - {1}}} {\text{d}}^{{ - {1}}} } \right) = \left( {{\text{TL}}_{{\text{D}}} {-}{\text{TL}}_{0} } \right)/\left( {{\text{t}}_{{\text{L}}} - {\text{t}}_{{\text{E}}} } \right)$$

where L_0_ and L_D_ indicate the total lipids (mg L^-1^) at the first day of cultivation (T_0_) and the desired days phase (T_D_), respectively.

#### Fatty acids methyl ester (FAMEs) determination

The lipid extracts were trans-esterified prior to gas chromatography analysis to fatty acid methyl esters (FAMEs)^78^. A DB-WAX column (30 m × 250 m internal diameter and 0.25 m film thickness) was installed in the GC. The constituents were identified by comparing the spectrum stored in the Wiley and NIST Mass Spectral Library data to the spectrum fragmentation pattern of the algal extracts.

#### Assessment of some biodiesel properties

The microalgae FAME composition using to calculate the biodiesel properties, incorporating unsaturation degree (Du), specific gravity (SG), kinematic viscosity (KV), cloud point (CP), cetane number (CN), iodine value (IV), saponification value (SV), higher heating value (HHV), were calculated from fatty acid profile according to Francisco et al.^79^, and Song et al.^80^ using the following Eqs. (3–10). Long chain saturation factor (LCSF, %wt.), and cold filter plugging point (CFPP, C) were determined in accordance with Ramos et al.^81^ using the following Eqs. (11–12).

3 $$Du = \sum C_{ = } \times Mf$$

where *C*_=_ refers to the number of C=C double bonds in fatty acid, while Mf represents mass fraction of each individual fatty acid.

4 $${\text{KV }}\left( {{\text{mm}}^{{2}} {\text{s}}^{{ - {1}}} } \right) = - 0.{\text{6316 Du}} + {2}0{65}$$

5 $${\text{SG }}\left( {{\text{kg}}^{{ - {1}}} } \right) = 0.00{\text{55 Du}} + 0.{8726}$$

6 $${\text{CP }}(^\circ {\text{C}}) = - {13}.{\text{356 Du}} + {19}.{994}$$

7 $${\text{CN}} = - {6}.{\text{6684 Du}} + {62}.{876}$$

8 $${\text{IV }}\left( {{\text{g}}/{\text{I}}_{{2}} /{1}00{\text{ g oil}}} \right) = {74}.{\text{373 Du}} + {12}.{71}$$

9 $${\text{SV}} = \sum {\left( {{56}0{\text{N}}} \right)/{\text{M}}}$$

In the previous equations, **D** is referred to the number of double bonds in the fatty ester, **M** is the molecular weight of the fatty ester, and **N** is the percentage of the certain fatty ester in the oil sample.

10 $${\text{HHV }}\left( {{\text{MJkg}}^{{ - {1}}} } \right) = {1}.{76}0{\text{1Du}} + {38}.{534}$$

11 $${\text{LCSF }}\left( {{\text{wt }}\% } \right) = \left( {0.{1} \times {\text{C}}_{{{16}}} } \right) + \left( {0.{5} \times {\text{C}}_{{{18}}} } \right) + \left( {{1}.0 \times {\text{C}}_{{{2}0}} } \right) + \left( {{1}.{5} \times {\text{C}}_{{{22}}} } \right) + \left( {{2}.0 \times {\text{C}}_{{{24}}} } \right)$$

12 $${\text{CFPP}}\left( {^\circ {\text{C}}} \right) = \left( {{3}.{1417} \times {\text{LCSF}}} \right) - {16}.{477}$$

In the above Eq. (11), C16, C_18_, C_20_, C_22_, and C_24_ refer to mass fraction of saturated fatty acids containing C16, C18, C20, C22, and C24, respectively.

### Statistical analysis

The experiments were carried out in triplicate and the results are shown as means standard deviation (± SD). Statistical analyses were carried out by using SPSS (version 23). One-way analysis (ANOVA) was used to compare mean values and to determine whether the results were statistically significant at probability levels ≤ 0.05, the differences were significant the post-hoc Dunn's multiple range test was used.

## Data Availability

All datasets are presented in the main manuscript.

## References

[1] Touliabah, H. E. S. & Almutairi, A. W. Effect of phytohormones supplementation under nitrogen depletion on biomass and lipid production of *Annochloropsis oceanica* for Integrated application in nutrition and biodiesel. *Sustainability* **13**(2), 592. 10.3390/su13020592 (2021). 10.3390/su13020592

[2] El-Sheekh, M. M., Mansour, H. M., Bedaiwy, M. Y. & Elshenoudy, R. A. Influence of nutrient supplementation and stress conditions on the biomass and lipid production of *Microchloropsis salina* for biodiesel production. *Biomass Convers. Biorefin.* **12**(11), 1–11. 10.1007/s13399-022-03434-9 (2022). 10.1007/s13399-022-03434-9

[3] Goldemberg, J. World Energy Assessment, Preface. United Nations Development Programme, New York, NY, USA. (2000).

[4] Kulkarni, M. G. & Dalai, A. K. Waste cooking iol-an economical source for biodiesel: A review. *Ind. Eng. Chem. Res.* **45**, 2901–2913 (2006). 10.1021/ie0510526

[5] Klass, L. D. *Biomass for Renewable Energy, Fuels and Chemicals* 1–2 (Academic Press, 1998).

[6] Abdelsalam, I. M., Elshobary, M., Eladawy, M. M. & Nagah, M. Utilization of multi-tasking non-edible plants for phytoremediation and bioenergy source—a review. *Phyton* **88**, 69–90. 10.32604/phyton.2019.06831 (2019). 10.32604/phyton.2019.06831

[7] Huete-Ortega, M. *et al.* Effect of ammonium and high light intensity on the accumulation of lipids in *Nannochloropsis oceanica* (CCAP 849/10) and *Phaeodactylum tricornutum* (CCAP 1055/1). *Biotechnol. Biofuels.* **11**, 60. 10.1186/s13068-018-1061-8 (2018). 29541157 10.1186/s13068-018-1061-8 PMC5844138

[8] Almutairi, A. W., El-Sayed, A. E. & Reda, M. M. Combined effect of salinity and pH on lipid content and fatty acid composition of *Tisochrysis lutea*. *Saudi J. Biol. Sci.* **27**, 3553–3558. 10.1016/j.sjbs.2020.07.027 (2020). 33304166 10.1016/j.sjbs.2020.07.027 PMC7714971

[9] Rodolfi, L. *et al.* Microalgae for oil: Strain selection, induction of lipid synthesis and outdoor mass cultivation in a low-cost photobioreactor. *Biotechnol. Bioengin.* **102**(1), 100–112. 10.1002/bit.22033 (2009). 10.1002/bit.22033 18683258

[10] Holbrook, G. P. *et al.* Use of the microalga Monoraphidium sp grown in wastewater as a feedstock for biodiesel: Cultivation and fuel characteristics. *Appl. Energy.* **131**, 386–393. 10.1016/j.apenergy.2014.06.043 (2014). 10.1016/j.apenergy.2014.06.043

[11] Bharte, S. & Desai, K. The enhanced lipid productivity of *Chlorella minutissima* and *Chlorella pyrenoidosa* by carbon coupling nitrogen manipulation for biodiesel production. *Environ. Sci. Pollut. Res.* **26**, 3492–3500 (2019). 10.1007/s11356-018-3757-5 30519914

[12] Hu, Q. Environmental effects on cell composition. In Richmond, A. & Hu, Q. (Eds.) Handbook of Microalgal Culture: Applied Phycology and Biotechnology. 2nd ed. Wiley Blackwell, West Sussex. 114–122 (2013).

[13] Hakalin, N. L. S., Paz, A. P., Aranda, D. A. G. & Moraes, L. M. P. Enhancement of cell growth and lipid content of a freshwater microalga *Scenedesmus* sp. by optimizing nitrogen phosphorus and vitamin concentrations for biodiesel production. *Nat. Sci.* **6**(12), 1044–1054. 10.4236/ns.2014.612095 (2006). 10.4236/ns.2014.612095

[14] Xu, H. X., Weng, X. Y. & Yang, Y. Effect of phosphorus deficiency on the photosynthetic characteristics of rice plants. *Russ. J. Plant. Physiol.* **54**(6), 741–748. 10.1134/S1021443707060040 (2007). 10.1134/S1021443707060040

[15] Almutairi, A. W. Effects of nitrogen and phosphorus limitations on fatty acid methyl esters and fuel properties of *Dunaliella salina*. *Environ. Sci. Pollut. Res.* **27**, 32296–32303. 10.1007/s11356-020-08531-8 (2020). 10.1007/s11356-020-08531-8 32242318

[16] Yodsuwan, N., Sawayama, S. & Sirisansaneeyakul, S. Effect of nitrogen concentration on growth, lipid production and fatty acid profiles of the marine diatom *Phaeodactylum tricornutum*. *Agric. Nat. Resour.* **51**, 190–197. 10.1016/j.anres.2017.02.004 (2017). 10.1016/j.anres.2017.02.004

[17] Pal, D., Khozin-Goldberg, I., Cohen, Z. & Boussiba, S. The effect of light, salinity, and nitrogen availability on lipid production by *Nannochloropsis* sp.. *Appl. Microbiol. Biotechnol.* **90**, 1429–1441. 10.1007/s00253-011-3170-1 (2011). 21431397 10.1007/s00253-011-3170-1

[18] Guschina, I. A. & Harwood, J. L. Lipids and lipid metabolism in eukaryotic algae. *Prog. Lipid Res.* **45**, 160–186 (2006). 16492482 10.1016/j.plipres.2006.01.001

[19] Gopinath, A., Puhan, S. & Nagarajan, G. Effect of biodiesel structural configuration on its ignition quality. *Int. J. Energy Environ.* **1**, 295–306 (2010).

[20] Knothe, G. Improving biodiesel fuel properties by modifying fatty ester composition. *Energy Environ. Sci.* **2**, 759–766 (2009). 10.1039/b903941d

[21] Nascimento, I. A. *et al.* Screening microalgae strains for biodiesel production: lipid productivity and estimation of fuel quality based on fatty acids profiles as selective criteria. *Bioenerg. Res.* **6**, 1–13. 10.1007/s12155-012-9222-2 (2013). 10.1007/s12155-012-9222-2

[22] Atabani, A. E. *et al.* A comprehensive review on biodiesel as an alternative energy resource and its characteristics. *Renew. Sust. Energ. Rev.* **16**(4), 2070–2093. 10.1016/j.rser.2012.01.003 (2012). 10.1016/j.rser.2012.01.003

[23] Yang, L., Chen, J. & Qin, S. Growth and lipid accumulation by different nutrients in the microalga *Chlamydomonas reinhardtii*. *Biotechnol. Biofuels.* **11**, 40. 10.1186/s13068-018-1041-z (2018). 29456627 10.1186/s13068-018-1041-z PMC5809890

[24] Pandit, P. R., Fulekar, M. H. & Karuna, M. S. L. Effect of salinity stress on growth, lipid productivity, fatty acid composition, and biodiesel properties in *Acutodesmus obliquus* and *Chlorella vulgaris*. *Environ. Sci. Pollut. Res.* **24**, 13437–13451. 10.1007/s11356-017-8875-y (2017). 10.1007/s11356-017-8875-y 28386901

[25] El-Sheekh, M., Abomohra, A. & Hanelt, D. Optimization of biomass and fatty acid productivity of *Scenedesmus obliquus* as a promising microalga for biodiesel production. *World J. Microbiol. Biotechnol.* **29**, 915–922 (2012). 23269508 10.1007/s11274-012-1248-2

[26] Marey, R. S., Abo-Shady, A. M., Khairy, H. M., Abd El-Moneim, A. M. & Abomohra, A. Enhanced lipid production and essential ω-fatty acids synthesis by the hypersaline biodiesel-promising microalga *Tetraselmis elliptica* through growth medium optimization. *Biomass Convers. Biorefin.*10.1007/s13399-022-03290-7 (2022). 10.1007/s13399-022-03290-7

[27] Alsull, M. & Omar, W. M. W. Responses of *Tetraselmis* sp. and *Nannochloropsis* sp. isolated from Penang National Park Coastal Waters, Malaysia, to the combined influences of salinity, light and nitrogen limitation. In: In International Conference on Chemical, Appl Ecol. Environ. Sci. (2012).

[28] Modiri, S. *et al.* Lipid production and mixotrophic growth features of cyanobacterial strains isolated from various aquatic sites. *Microbiology* **161**(3), 662–673. 10.1099/mic.0.000025 (2015). 25575545 10.1099/mic.0.000025

[29] Ashour, M., Elshobary, M. E., El-Shenody, R., Kamil, A. W. & Abomohra, A. Evaluation of a native oleaginous marine microalga *Nannochloropsis oceanica* for dual use in biodiesel production and aquaculture feed. *Biomass Bioenergy* **120**, 439–447. 10.1016/j.biombioe.2018.12.009 (2018). 10.1016/j.biombioe.2018.12.009

[30] Fawzy, M. A., El-Otify, A. M., Adam, M. S. & Moustafa, S. A. The impact of abiotic factors on the growth and lipid accumulation of some green microalgae for sustainable biodiesel production. *Environ. Sci. Pollut. Res.* **28**, 42547–42561. 10.1007/s11356-021-13781-1 (2021). 10.1007/s11356-021-13781-1 33813694

[31] Elshobary, M. E. *et al.* Influence of nutrient supplementation and starvation conditions on the biomass and lipid productivities of *Micractinium reisseri* grown in wastewater for biodiesel production. *J. Environ. Manag.* **250**, 109529. 10.1016/j.jenvman.2019.109529 (2019). 10.1016/j.jenvman.2019.109529 31518792

[32] Xin, L., Ying, H. H., Ke, G. & Xue, S. Y. Effects of different nitrogen and phosphorus concentrations on the growth nutrient uptake and lipid accumulation of a freshwater microalga *Scenedesmus* sp.. *Bioresour. Technol.* **101**, 5494–5500. 10.1016/j.biortech.2010.02.016 (2010). 20202827 10.1016/j.biortech.2010.02.016

[33] Kamalanathan, M., Pierangelini, M., Shearman, L. A., Gleadow, R. & Beardall, J. Impacts of nitrogen and phosphorus starvation on the physiology of *Chlamydomonas reinhardtii*. *J. Appl. Phycol.* **28**, 1509–1520. 10.1007/s10811-015-0726-y (2001). 10.1007/s10811-015-0726-y

[34] Qiao, T., Zhao, Y., Zhong, D. B. & Yu, X. Hydrogen peroxide and salinity stress act synergistically to enhance lipids production in microalga by regulating reactive oxygen species and calcium. *Algal. Res.* **53**, 102017 (2021). 10.1016/j.algal.2020.102017

[35] Salama, E. S. *et al.* Biomass, lipid content, and fatty acid composition of freshwater *Chlamydomonas mexicana* and *Scenedesmus obliquus* grown under salt stress. *Bioprocess Biosyst. Eng.* **36**, 827–833. 10.1007/s00449-013-0919-1 (2013). 23411874 10.1007/s00449-013-0919-1

[36] Osman, M. E. H., Abo-Shady, A. M., Gheda, S. F., Desoki, S. M. & Elshobary, M. E. Unlocking microalgae cultivated on wastewater combined with salinity stress to improve biodiesel production. *Environ. Sci. Pollut. Res.* **30**, 114610–114624. 10.1007/s11356-023-30370-6 (2023). 10.1007/s11356-023-30370-6 PMC10663198 37863854

[37] Chandra, R., Amit, & Ghosh, U. K. Effects of various abiotic factors on biomass growth and lipid yield of *Chlorella minutissima* for sustainable biodiesel production. Environ. Sci. Pollut. Res. 26(4), 3848–3861 (2019). 10.1007/s11356-018-3696-1. 10.1007/s11356-018-3696-1 30539390

[38] Breuer, G., Lamers, P. P., Martens, D. E., Draaisma, R. B. & Wijffels, R. H. The impact of nitrogen starvation on the dynamics of triacylglycerol accumulation in nine microalgae strains. *Bioresour. Technol.* **124**, 217–226. 10.1016/j.biortech.2012.08.003 (2012). 22995162 10.1016/j.biortech.2012.08.003

[39] Hounslow, E. *et al.* Quantitative proteomic comparison of salt stress in *Chlamydomonas reinhardtii* and the snow alga *Chlamydomonas nivalis* reveals mechanisms for salt-triggered fatty acid accumulation via reallocation of carbon resources. *Biotechnol. Biofuels.* **14**, 1–25 (2021). 34022944 10.1186/s13068-021-01970-6 PMC8141184

[40] Fal, S., Aasfar, A., Rabie, R., Smouni, A. E. L. & Arroussi, H. Salt induced oxidative stress alters physiological, biochemical and metabolomic responses of green microalga *Chlamydomonas reinhardtii*. *Heliyon.* **8**(1), 8811. 10.1016/j.heliyon.2022.e08811 (2022). 10.1016/j.heliyon.2022.e08811 PMC8792077 35118209

[41] Ji, X. *et al.* The effect of NaCl stress on photosynthetic efficiency and lipid production in freshwater microalga—*Scenedesmus obliquus* XJ002. *Sci. Total Environ.* **633**, 593–599 (2018). 29587228 10.1016/j.scitotenv.2018.03.240

[42] Khan, M. I., Shin, J. H. & Kim, J. D. The promising future of microalgae: Current status, challenges, and optimization of a sustainable and renewable industry for biofuels, feed, and other products. *Microb. Cell Fact.* **17**, 36. 10.1186/s12934-018-0879-x (2018). 29506528 10.1186/s12934-018-0879-x PMC5836383

[43] El Shafay, S. M., Gaber, A., Alsanie, W. F. & Elshobary, M. E. Influence of nutrient manipulation on growth and biochemical constituent in *Anabaena variabilis* and *Nostoc muscorum* to enhance biodiesel production. *Sustainability* **13**, 9081. 10.3390/su13169081 (2021). 10.3390/su13169081

[44] Harwati, T. U., Willke, T. & Vorlop, K. D. Characterization of the lipid accumulation in a tropical freshwater microalgae *Chlorococcum* sp. *Bioresou. Technol.* **121**, 54–60. 10.1016/j.biortech.2012.06.098 (2012). 10.1016/j.biortech.2012.06.098 22858468

[45] George, B., Pancha, I., Desai, C., Chokshi, K. & Paliwal, C. Effects of different media composition, light intensity and photoperiod on morphology and physiology of freshwater microalgae *Ankistrodesmus falcatus*–a potential strain for bio-fuel production. *Bioresou. Technol.* **171**, 367–374 (2014). 10.1016/j.biortech.2014.08.086 25218209

[46] Sajjadi, B., Chen, W. Y., Raman, A. A. A. & Ibrahim, S. Microalgae lipid and biomass for biofuel production: A comprehensive review on lipid enhancement strategies and their effects on fatty acid composition. *Renew. Sustain. Energy Rev.* **97**, 200–232. 10.1016/j.rser.2018.07.050 (2018). 10.1016/j.rser.2018.07.050

[47] Mirizadeh, S., Nosrati, M. & Shojaosadati, S. A. Synergistic effect of nutrient and salt stress on lipid productivity of *Chlorella vulgaris* through two-stage cultivation. *Bioenergy Res.* **13**, 507–517 (2020). 10.1007/s12155-019-10077-8

[48] Juneja, A., Ceballos, R. M. & Murthy, G. S. Effects of environmental factors and nutrient availability on the biochemical composition of algae for biofuels production: A review. *Energies* **6**(9), 4607–4638. 10.3390/en6094607 (2013). 10.3390/en6094607

[49] Hang, L. T., Mori, K., Tanaka, Y., Morikawa, M. & Toyama, T. Enhanced lipid productivity of *Chlamydomonas reinhardtii* with combination of NaCl and CaCl_2_ stresses. *Bioproc. Biosyst.* **43**, 971–980. 10.1007/s00449-020-02293-w (2020). 10.1007/s00449-020-02293-w 32008095

[50] Chen, B. *et al.* Manipulating environmental stresses and stress tolerance of microalgae for enhanced production of lipids and value-added products–a review. *Bioresour. Technol.* **244**, 1198–1206. 10.1016/j.05.170 (2017). 28601395 10.1016/j.05.170

[51] Dahmen, M. B. I., Chtourou, H., Rezgui, F., Sayadi, S. & Dhouib, A. Salinity stress increases lipid, secondary metabolites and enzyme activity in *Amphora subtropica* and *Dunaliella* sp. for biodiesel production. *Bioresour. Technol.* **244**, 816–825. 10.1016/j.biotech.2016.07.022 (2016). 10.1016/j.biotech.2016.07.022 27428298

[52] Zaki, M. *et al.* Potential applications of native cyanobacterium isolate (*Arthrospira platensis* NIOF17/003) for biodiesel production and utilization of its byproduct in marine rotifer (*Brachionus plicatilis*) production. *Sustainability* **13**, 1769. 10.3390/su13041769 (2021). 10.3390/su13041769

[53] Sukkrom, K., Bunnag, B. & Pavasant, P. Study of increasing lipid production from reused medium for *Ankistrodesmus* sp. culture. *3rd Int. Conf. Inform. Environ. Energy Appl. IPCBEE.* **66**, 41–45 (2014).

[54] Widianingsih, H. R., Endrawati, H. & Mamuaja, J. Fatty acid composition of marine microalgae in Indonesia. *J. Trop. Biol. Conserv.* **10**, 75–82 (2013).

[55] Srinuanpan, S., Cheirsilp, B., Prasertsan, P., Kato, Y. & Asano, Y. Strategies to increase the potential use of oleaginous microalgae as biodiesel feedstocks: Nutrient starvations and cost-effective harvesting process. *Renew. Energy* **122**, 507–516. 10.1016/j.renene.2018.01.121 (2018). 10.1016/j.renene.2018.01.121

[56] Zhang, Y., Gao, W., Lv, Y., Bai, Q. & Wang, Y. Exogenous melatonin confers salt stress tolerance to *Chlamydomonas reinhardtii* (Volvocales, Chlorophyceae) by improving redox homeostasis. *Phycologia* **57**, 680–691. 10.2216/17-141.1 (2018). 10.2216/17-141.1

[57] Mata, T. M., Martins, A. & Caetano, N. Microalgae for biodiesel production and other applications: A review. *Renew. Sustain. Energy Rev.* **14**, 217–232. 10.1016/j.rser.07.020 (2010). 10.1016/j.rser.07.020

[58] Cepák, V., Přibyl, P., Kohoutková, J. & Kaštánek, P. Optimization of cultivation conditions for fatty acid composition and EPA production in the Eustigmatophycean Microalga *Trachydiscus Minutus*. *J. Appl. Phycol.* **26**, 181–190. 10.1007/s10811-013-0119-z (2014). 10.1007/s10811-013-0119-z

[59] Shekh, A. Y. *et al.* Biomass and lipid enhancement in *Chlorella* sp. with emphasis on biodiesel quality assessment through detailed FAME signature. *Bioresour. Technol.* **201**, 276–286. 10.1016/j.2015.11.058 (2016). 26679050 10.1016/j.2015.11.058

[60] Hu, Q., Sommerfeld, M., Jarvis, E. & Ghirardi, M. Microalgal triacylglycerols as feedstocks for biofuel production: Perspectives and advances. *Plant J.* **54**(4), 621–639. 10.1111/j.1365-313X.2008.03492.x (2008). 18476868 10.1111/j.1365-313X.2008.03492.x

[61] Karpagam, R., Preeti, R. & Ashokkumar, B. Enhancement of lipid production and fatty acid profiling in *Chlamydomonas reinhardtii,* CC1010 for biodiesel production. *Ecotoxicol. Environ. Saf.* **121**, 253–257. 10.1016/j.ecoenv.2015.03.015 (2015). 25838071 10.1016/j.ecoenv.2015.03.015

[62] Okiemen, F. E. & Omosigho, H. N. On the fuel properties of methyl esters of palm kernel oil. *Niger. J. Appl. Sci.* **26**, 90–94 (2008).

[63] Ma, Y. *et al.* Increased lipid productivity and TAG content in *Nannochloropsis* by heavy-ion irradiation mutagenesis. *Bioresour. Technol.* **136**, 360–367. 10.1016/j.biortech.2013.03.020 (2013) (**Epub 2013 Mar 14 PMID: 23567703**). 23567703 10.1016/j.biortech.2013.03.020

[64] Igbum, O. G., Leke, L., Okoronkwo, M. U., Eboka, A. & Nwadinigwe, C. A. Evaluation of fuel properties from free fatty acid compositions of methyl esters obtained from four tropical virgin oils. *Int. J. Appl. Chem.* **9**(1), 37–49 (2013).

[65] Lapuerta, M., Sanz-Argent, J. & Robert, R. Ignition Characteristics of diesel fuel in a constant volume bomb under diesel-like conditions effect of the operation parameters. *Energy Fuels* **28**(8), 5445–5454 (2014). 10.1021/ef500535j

[66] Neupane, D., Kafle, S., Karki, K. R., Kim, D. H. & Pradhan, P. Solar and wind energy potential assessment at provincial level in Nepal: Geospatial and economic analysis. *Renew. Energy.* **181**, 278–291 (2022). 10.1016/j.renene.2021.09.027

[67] Rai, V., Muthuraj, M., Gandhi, M. N., Das, D. & Srivastava, S. Real-time iTRAQ-based proteome profiling revealed the central metabolism involved in nitrogen starvation induced lipid accumulation in microalgae. *Sci. Rep.* **7**, 1–16 (2017). 28378827 10.1038/srep45732 PMC5381106

[68] Anahas, A. M. P. & Muralitharan, G. Characterization of heterocystous cyanobacterial strains for biodiesel production based on fatty acid content analysis and hydrocarbon production. *Energy Convers. Manag.* **157**, 423–437. 10.1016/j.enconman.2017.12.012 (2018). 10.1016/j.enconman.2017.12.012

[69] Mandotra, S. K., Kumar, P., Suseela, M. R., Nayaka, S. & Ramteke, P. W. Evaluation of fatty acid profile and biodiesel properties of microalga *Scenedesmus abundans* under the influence of phosphorus, pH and light intensities. *Bioresour. Technol.* **201**, 222–229. 10.1016/j.biortech.2015.11.042 (2016). 26675046 10.1016/j.biortech.2015.11.042

[70] Anahas, A. M. P. & Muralitharan, G. Isolation and screening of heterocystous cyanobacterial strains for biodiesel production by evaluating the fuel properties from fatty acid methyl ester (FAME) profiles. *Bioresour Technol.* **184**, 9–17 (2015). 25435067 10.1016/j.biortech.2014.11.003

[71] Morsi, H. H. *et al.* Screening the pollution-tolerant *Chlorococcum* sp. (Chlorophyceae) grown in municipal wastewater for simultaneous nutrient removal and biodiesel production. *Water* **15**, 1723. 10.3390/w15091723 (2023). 10.3390/w15091723

[72] Stein, J. R. Growth and mating of gonium pectorale (volvocales) in defined media 1. *J. Phycol.* **2**, 23–28 (1966). 27053021 10.1111/j.1529-8817.1966.tb04587.x

[73] Wetherel, D. F. Culture of fresh water algae in enriched natural sea water. *Physiol. Plantarum.* **14**(1), 1–6 (1961). 10.1111/j.1399-3054.1961.tb08131.x

[74] Abomohra, A., Wagner, M., El-Sheekh, M. & Hanelt, D. Lipid and total fatty acid productivity in photoautotrophic fresh water microalgae: Screening studies towards biodiesel production. *J. Appl. Phycol.* **25**, 931–936 (2013). 10.1007/s10811-012-9917-y

[75] Metzner, H., Rau, H. & Senger, H. Untersuchungen zur synchronisierbarkeit einzelner pigmentmangel-mutanten von *Chlorella*. *Planta* **65**, 186–194 (1965). 10.1007/BF00384998

[76] El-Sheekh, M., Abomohra, A., AbdEl-Azim, M. & Abou-Shanab, R. Effect of temperature on growth and fatty acids profile of the biodiesel producing microalga *Scenedesmus acutus*. *Biotechnol. Agron. Soc. Environ.* **21**(4), 233–239 (2017). 10.25518/1780-4507.15291

[77] Andrade, M. R. & Costa, J. A. V. Mixotrophic cultivation of microalga *Spirulina platensis* using molasses as organic substrate. *Aquaculture.* **264**, 130–134 (2007). 10.1016/j.aquaculture.2006.11.021

[78] Arif, M. *et al.* Microalgae isolation for nutrient removal assessment and biodiesel production. *Bioenergy Res.* **13**, 1247–1259 (2020). 10.1007/s12155-020-10136-5

[79] Francisco, E. C., Neves, D. B., Jacob-Lopes, E. & Franco, T. T. Microalgae as feedstock for biodiesel production: carbon dioxide sequestration, lipid production and biofuel quality. *J. Chem Technol. Biotechnol.* **85**(3), 395–403. 10.1002/jctb.2338 (2010). 10.1002/jctb.2338

[80] Song, M., Pei, H., Hu, W. & Ma, G. Evaluation of the potential of 10 microalgal strains for biodiesel production. *Bioresour. Technol.* **141**, 245–251 (2013). 23489572 10.1016/j.biortech.2013.02.024

[81] Ramos, M. J., Fernández, C. M., Casas, A., Rodríguez, L. & Pérez, Á. Influence of fatty acid composition of raw materials on biodiesel properties. *Bioresour. Technol.* **100**(1), 261–268. 10.1016/j.biortech.2008.06.039 (2009). 18693011 10.1016/j.biortech.2008.06.039

